# Supplementary material for: A Review of Gas Measurement Set-Ups
Source: Sensors (Basel). 2022 Mar 27;22(7):2557. doi: 10.3390/s22072557 (PMC9002727; doi:10.3390/s22072557)
Supplement: Supplementary file 1 [file sensors-22-02557-s001.zip › sensors-1638380-supplementary.pdf]

Table S1. Summary of gas sensing methods - their advantages, disadvantages and applications [130].

Gas Detection Technologies	Advantages	Disadvantages	Practical Consideration
Catalytic gas detectors	- Simple operation<br>- Robust and easy to use and calibrate<br>- Highly reliable<br>- Easily calibrated for individual gases such as hydrogen	- Frequent calibration required due to inactivity or contamination<br>- Prolonged exposure to flammable gas reduces sensitivity	- Catalytic sensors are generally helpful to detect gases like hydrogen where other point gas detectors are not as responsive.<br>- Sensor beads might have to be replaced or sensors have to be calibrated frequently to maintain high reliability.<br>- Calibration kits are available from different vendors to allow remote calibration since sensors could be located at heights not easily accessible.<br>- Power requirement of the catalytic sensors is not very high and generally operates on loop power from controller.<br>- Accuracy ranges from 3-5% depending on the %LFL (Lower Flammability Limit) range.<br>- Typical response time to 50% LFL is 10 seconds and to 90% LFL is 30 seconds. This is the time it would take for the sensors to detect the correct gas concentration and provide a signal once the gas is in contact with the sensor.<br>- It can operate over a wide temperature range, -40 ˚C to +75 ˚C.<br>- Very high reliability in environments with extreme temperatures, humidity and vibrations.
InfraRed (IR) Gas Detectors	- Most common gas detection system<br>- Wide choice of vendors and cost competitive<br>- Easy to setup and calibrate<br>- Calibration is not required as often as catalytic sensors<br>- Immunity to noise and contaminants<br>- Works continuously in presence of flammable gas without degradation	- Initial purchase and installation cost is high<br>- Gas has to be infrared active, such as hydrocarbons<br>- Not effective in extreme temperatures, humidity or high vibration environments<br>- Does not perform well for multiple gas applications	- IR sensors are generally helpful to detect hydrocarbons.<br>- Power requirement of IR sensors ranges from 5-20W. IR sensors typically operate on loop power from the controller.<br>- Accuracy ranges from 1-5% depending on the %LFL range it's measuring.<br>- Typical response time to 50% LFL is 5 seconds and to 90% LFL is 10 seconds.<br>- IR sensors can operate over a wide temperature range of -40 ˚C to +75 ˚C.<br>- IR sensors are calibrated to a particular gas, example methane or propane. If other gases are measured using the same sensor, vendors must provide adjustment curves to determine the concentration. Accuracy of such corrected measurements is limited.<br>- If the gas sensor becomes "saturated", it may require significant time for the sensor value to reduce to normal level after it has come in contact with the gas. This is especially true if a hydrophobic filter or a weather baffle is used.

			- Deviations in mounting from the manufacturer's recommended orientation may result in large errors in measured concentrations.
Open Path Detectors	- Extensively used in offshore platforms and on-shore facilities to detect gas release in a wider area.<br>- Used both as early alarms as well as to trigger evacuations.<br>- Needs less installed equipment than point detectors if the purpose is to only detect gas release and not the concentration of gas.	- Open path detectors are extremely sensitive to maintaining line of sight between transmitter and receiver.<br>- This makes initial commissioning very difficult and time consuming. They are very susceptible to temporary obstructions like rail cars, scaffolds, other equipment or vehicles. Notorious for nuisance alarms or trips.<br>- Unit does not report the value of %LFL, only gives the value of LFL-m.<br>- Initial cost of the instrument and installation is significantly higher than point IR detectors.<br>- Vibrations may result in misalignment of the source and receiver units.	- Line of Sight sensors are generally helpful to detect hydrocarbons. However, very few toxic units are available.<br>- Power requirements of the IR sensors range from 20-50W. Some units draw higher power if they don't need fine adjustments to ensure line of sight. These units constantly transmit IR beams over a wider area.<br>- If power is not an issue, these units could be considered to reduce calibration time.<br>- Accuracy ranges around 1% depending on the %LFL-m range it's measuring<br>- Typical response time to 90% LFL is 5 seconds.<br>- It can typically operate over a wide temperature range, -50 °C to +50 °C.<br>- Area detectors are not calibrated to a particular gas, so they can provide %LFL-m values for a range of hydrocarbon gases.<br>- Toxic detectors are calibrated to particular gases like hydrogen sulfide or ammonia and they should only be used in the applications for which they are designed.<br>- Alignment of the source/receiver requires significant time and effort. Units may become unaligned due<br>- to vibrations, weather, or accidental encounters.<br>- Even though these units do not need the gas to contact the sensors, correct placement is still critical for<br>- adequate detection. The gas cloud must still interact with the IR beam to trigger an alarm.
Acoustic Detectors	- Response time is negligible.<br>- Detects leaks independent of gas.<br>- Most acoustic units can be programmed to learn certain types of release gas based on historical data.<br>- This can help increase the accuracy of the measurement.	- Prone to nuisance alarms/trips if not configured correctly, since it can detect any leak. A nitrogen or instrument air leak in the area could trigger an alarm/trip.	- Acoustic technology for gas detection has developed a lot over the last few years; however, additional work is ongoing to reduce nuisance alarms/trips.<br>- Acoustic detectors may be best used as a preliminary alarm, with point or area detectors deployed to trigger any type of shutdown (either automatic or by an operator).<br>- Most of the acoustic units are battery operated and require low power (1-2W).<br>- Installation is fairly simple and costs significantly less than gas detectors. Proper placement for leak detection not as critical as with gas detectors.

	- It can typically operate over a wide temperature range, -50 ˚C to +75 ˚C.
